# Performance of centralized versus decentralized tuberculosis treatment services in Southern Brazil, 2006–2015

**DOI:** 10.1186/s12889-018-5468-8

**Published:** 2018-04-25

**Authors:** Mara Cristina Scheffer, Rodrigo IVAN Prim, Leticia Muraro Wildner, Taiane Freitas Medeiros, Rosemeri Maurici, Emil Kupek, Maria Luiza Bazzo

**Affiliations:** 1 Departamento de Análises Clínicas for Programa de Pós-Graduação em Farmácia da, Universidade Federal de Santa Catarina, Florianópolis, SC Brazil; 2 Departamento de Clínica Médica, Universidade Federal de Santa Catarina, Florianópolis, SC Brazil; 3 Departamento de Saúde Pública, Universidade Federal de Santa Catarina, Florianópolis, SC Brazil; 4 Laboratório de Biologia Molecular, Sorologia e Micobactérias, Departamento de Análises Clínicas, Centro de Ciências da Saúde, Universidade Federal de Santa Catarina, Campos Universitário- Trindade, Florianopolis, SC 88040-900 Brazil

**Keywords:** Tuberculosis, Public health, Treatment outcome, Decentralized

## Abstract

**Background:**

Tuberculosis (TB) control programs face the challenges of decreasing incidence, mortality rates, and drug resistance while increasing treatment adherence. The Brazilian TB control program recommended the decentralization of patient care as a strategy for combating the disease. This study evaluated the performance of this policy in an area with high default rates, comparing epidemiological and operational indicators between two similar municipalities.

**Methods:**

This study analyzed epidemiological and operational indicators on new cases of pulmonary tuberculosis reported in the Brazilian Notifiable Diseases Information System between 2006 and 2015. In addition, to characterize differences between the populations of the two studied municipalities, a prospective cohort study was conducted between 2014 and 2015, in which patients with new cases of culture-confirmed pulmonary tuberculosis were interviewed and monitored until the disease outcome. A descriptive analysis, the chi-square test, and a Poisson regression model were employed to compare TB treatment outcomes and health care indicators between the municipalities.

**Results:**

Two thousand three hundred nine cases were evaluated, of which 207 patients were interviewed. Over the 2006–2015 period, TB incidence per 100,000 population in the municipality with decentralized care was significantly higher (39%, 95% CI 27–49%) in comparison to that of the municipality with centralized care. TB treatment default rate (45%, 95% CI 12–90%) was also higher in the municipality with decentralized care. During the two-year follow-up, significant differences were found between patients in centralized care and those in decentralized care regarding treatment success (84.5 vs. 66.1%), treatment default (10.7 vs. 25.8%), illicit drug use (27.7 vs. 45.9%), and homelessness (3.6 vs. 12.9%). The operational indicators revealed that the proportion of control smear tests, medical imaging, and HIV tests were all significantly higher in the centralized care. However, a significantly higher proportion of patients started treatment in the early stages of the disease in the municipality with decentralized care.

**Conclusions:**

These data showed a low success rate in TB treatment in both municipalities. Decentralization of TB care, alone, did not improve the main epidemiological and operational indicators related to disease control when compared to centralized care. Full implementation of strategies already recommended is needed to improve TB treatment success rates.

## Background

Tuberculosis (TB) control programs aim to reduce disease mortality, prevalence, and incidence, focusing their actions on detection and treatment, especially of patients who are responsible for maintaining the transmission cycle of *Mycobacterium tuberculosis* [1]. The Directly Observed Treatment Short-Course (DOTS) strategy, recommended by the World Health Organization (WHO) since 1993, remains as the main pillar for TB control programs. This strategy was based on a model developed by Karel Styblo in 1970, using a management approach for case detection and treatment through supplying basic health units (BHUs) with the necessary resources and personnel to perform the diagnosis, treatment, recording, and progress reporting of TB patients in areas with 100,000 to 150,000 inhabitants [2]. The recommendations of the DOTS strategy were established based on observations made of the health services and the acknowledgement that their lack of organization to ensure the detection and cure of TB patients was a major problem in TB control [2, 3]. Other shortcomings also involved the access to treatment, high default rates among patients, and the absence of effective monitoring and surveillance systems [3].

The WHO target of achieving 85% of cure employing the DOTS strategy was increased to 95% in 2016 with the “End TB Strategy” [2, 4]. Brazilian government opted to decentralize tuberculosis control programs by expanding patient care to the primary health network instead of offering TB treatment in specialized municipal outpatient clinics [5]. However, the process of decentralization has not been adopted by all Brazilian municipalities designated as priorities by the National Tuberculosis Control Program (PNCT).

The objective of the present study was to compare the performance of tuberculosis control programs in two municipalities with similar demographic and socioeconomic characteristics and different models of TB care. To evaluate the effectiveness and quality of health care, this study analyzed epidemiological indicators [6] (related to tuberculosis control) and operational indicators [6] (related to patient care), comparing data from Florianópolis, a municipality with 100% decentralized TB service, to data from São José, a municipality that opted in keeping TB patient care centralized on a specialized clinic.

## Methods

This observational descriptive study evaluated patients under treatment for pulmonary tuberculosis in the municipalities of Florianópolis and São José, using two data sources, each analyzing a different period. Data on a 10-year period (2006 to 2015) were retrieved from the Notifiable Diseases Information System (SINAN), available on the TABNET platform of the Santa Catarina Epidemiological Surveillance Department (DIVE/SC), and a prospective cohort study was performed with patients diagnosed with culture-confirmed pulmonary tuberculosis who started treatment between 2014 and 2015.

### Characterization of the municipalities

Two priority municipalities, according to the PNCT, were selected for this study. Municipality of Florianópolis, SC: 469,690 inhabitants (2015), territorial area of 675,409km^2^, population density of 623.68 inhabitants/km^2^, Municipal Human Development Index (MHDI) of 0.847, gross domestic product (GDP) per capita of 32,385.04 Brazilian reais. Municipality of São José, SC: 232,309 inhabitants (2015), territorial area of 150,453 km^2^, population density 1376.78 inhabitants/km^2^, MHDI of 0.809, GDP per capita of 34,181.78 Brazilian reais. Florianópolis has extensive conservation areas. Considering only urban areas, the population density of both municipalities is similar. Care decentralization in these municipalities of Santa Catarina was preceded by the training of health care professionals by the State Epidemiological Surveillance Service with the objective of preparing BHU personnel for the diagnosis, treatment, follow-up, and notification of TB cases. In Florianópolis, as of 2006, the primary health care network is responsible for the decentralized treatment of TB, and patients are assisted by a multidisciplinary health team in 70 BHUs. In São José, a specialized outpatient clinic was responsible for the centralized treatment of the disease during the 2012–2016 period, and all patients were seen by a pulmonologist. However, TB treatment was decentralized in the preceding years (2006–2011). In both municipalities, the directly observed treatment has not been performed routinely and systematically, being restricted to cases of MDR-TB and cases of treatment failure.

### Studied indicators (2006 to 2015)

This study evaluated the main epidemiological and operational indicators available on the TABNET/DIVE platform between 2006 and 2015. The analyzed epidemiological indicators of disease control were TB and pulmonary TB incidence rates and treatment completion and default rates among new cases. The analyzed operational indicators included the proportion of TB cases confirmed by culture, sputum smear microscopy in the second month of therapy, use of radiological resources for diagnosis and follow-up of lesion evolution, and HIV testing.

### Studied population (2014 and 2015)

To verify the occurrence of systematic errors due to differences between the TB-positive populations of both municipalities, a characterization of these populations was performed. This was accomplished through a prospective cohort of patients from both municipalities, who had a culture-confirmed tuberculosis diagnosis and started treatment with the basic regimen (rifampicin, isoniazid, pyrazinamide, and ethambutol) between 2014 and 2015. The following variables were evaluated: gender, level of schooling, cigarette smoking habits, abusive alcohol consumption, illicit drug use, proportion of patients experiencing homelessness, and TB/HIV co-infection. After signing the informed consent form, patients were interviewed and monitored through their medical records until the outcome of the disease (treatment success or default, death, or transfer to another jurisdiction). There was no interference in the patients’ routine, and treatments followed the PNCT guidelines. This study was approved by the UFSC Human Health Research Ethics Committee (protocol no. 550.598/2014).

### Studied indicators (2014 and 2015)

Two TB-related indicators unavailable in the Information System (TABNET/DIVE) were evaluated: the time elapsed between the beginning of symptoms and the initiation of treatment, and the place of TB diagnosis.

### Data management and analysis

New cases were defined as those in which patients had never received anti-TB treatment or had been treated for less than 30 days [7]; Treatment outcomes were classified according to the following criteria: treatment success, the sum of cured and treatment completed cases [8]; and default, treatment interrupted for one consecutive month or more [7]. Data were analyzed using the SPSS 22.0® software (SPSS Inc., Chicago, USA) and summarized as absolute numbers and/or percentages. Associations between the variables and the outcomes were assessed using the chi-square test and the Fischer’s exact test, with a 5% significance level. Additionally, the comparison between centralized and decentralized care over the 2006–2015 period was calculated using a Poisson regression model with 95% confidence interval to estimate relative risks. An indicator variable was added to the regression model to account for the 2006–2011 period of decentralized TB care in São José.

## Results

Between 2006 and 2015, Florianópolis notified 2316 new TB cases, of which 1706 were pulmonary TB. Over the same period, São José notified 843 new TB cases, of which 603 were pulmonary TB. Table 1 lists the indicators related to tuberculosis control and patient care over a 10-year period in both municipalities. The incidence of tuberculosis, pulmonary tuberculosis, and the rate of treatment default were significantly higher (38%, 39%, and 45%, respectively) in Florianópolis than in São José. The percentage of defaulted cases decreased in the years of 2010 and 2011 in São José, remained constant in the following years, reached its worst performance in 2014, and improved slightly in 2015. Treatment default rates were very high throughout the entire studied period in Florianópolis, except in the year of 2006. There was a great variation in treatment success rates, which in Florianópolis reached their worst performance in 2008 (57.3%), slowly improving through the years until 2012 (74.1%), then falling again, and closing the period of observation at 58.6%. In the municipality of São José, treatment success had its worst performance in the same year (2008 = 62.3%) as in the municipality of Florianópolis, reaching 78.0% in 2010, falling again in the following years, and closing the period of observation with a discreet increase (70.3%). In Florianópolis, the proportion of culture-confirmed diagnosis was significantly higher (40%) than in São José. However, other indicators related to patient care, such as performing sputum smear examination in the second month of therapy, imaging exams, and HIV testing, were significantly higher in São José than in Florianópolis.

**Table 1:** Comparison of epidemiological and operational indicators related to tuberculosis over a 10-year period in the municipalities of Florianópolis (non-decentralized care in Basic Health Units) and São José (decentralized care - 2006-2011 and centralized care - 2012-2015 in a specialized outpatient clinic), in the Southern region of Brazil, 2006–2015

Indicator	Municipality	2006	2007	2008	2009	2010	2011	2012	2013	2014	2015	Mean	RR (95% CI)^a^
TB incidence/100.000 population	São José	39	32	37	30	37	29	39	38	46	27	35.4	1.38 (1.27–1.49)^b^
	Florianópolis	45	42	46	49	45	51	50	57	53	48	44.1	
PTB incidence/100.000 population	São José	29	20	27	23	29	19	26	31	33	18	25.5	1.39 (1.27–1.53)^b^
	Florianópolis	34	29	32	36	28	34	38	42	40	40	35.3	
Proportion of treatment completion in PTB NC	São José	64.4	72.5	62.3	71.4	78.0	70.1	63.1	65.2	62.7	70.3	68.1	0.97 (0.87–1.09)
	Florianópolis	72.6	58.8	57.3	60.9	68.1	70.8	74.1	65.1	68.3	58.6	65.5	
Proportion of treatment default in NC of PTB	São José	10.2	10	9.4	10.2	5.1	4.9	10.5	13.0	15.7	5.4	9.4	1.45 (1.12–1.90)^b^
	Florianópolis	7.2	20.2	18.1	13.0	16.8	14.6	10.2	13.5	17.3	15.9	14.7	
Proportion of culture-confirmed PTB NC	São José	39	17.5	30.2	20.4	39	26.8	40.3	43.5	44.7	34.1	33.5	1.40 (1.22–1.61)^b^
	Florianópolis	22.3	29.4	26.8	35.6	58	64.6	61.4	58.8	63.6	63.6	48.4	
Proportion of smear testing in the 2nd month of therapy in NC of PTB	São José	–	52.5	50.9	49.0	78.0	65.8	61.4	63.8	82.9	–	63.0	0.76 (0.66–0.88)^b^
	Florianópolis	–	49.6	44.2	56.9	53.8	44.4	42.2	45.8	57.1	–	49.2	
Proportion of imaging exams in NC of PTB	São José	98.4	95	92.5	93.9	100	95.1	96.5	92.8	90.8	100	95.5	0.75 (0.68–0.84)^b^
	Florianópolis	77.6	81.7	75.8	77.2	71.3	71.8	68.0	71.0	68.0	61.3	72.3	
Proportion of HIV testing in NC of PTB	São José	91.5	87.5	92.5	95.9	96.6	100	96.5	100	98.7	97.6	95.7	0.74 (0.67–0.82)^b^
	Florianópolis	69.8	69.0	69.6	69.2	73.1	72.9	78.3	74.5	72.3	62.2	71.1	
Proportion of TB/HIV co-infection in NC of PTB	São José	20.3	17.5	32.1	26.5	27.1	12.2	12.3	23.2	27.6	24.4	22.3	0.93 (0.76–1.14)
	Florianópolis	23.7	25.2	29.0	25.3	19.3	20.8	18.7	21.3	16.8	16.0	21.6	

*TB* all types of tuberculosis, *PTB* pulmonary tuberculosis, *NC* new cases

^a^Florianópolis in comparison to São José

^b^*p* < 0.05

In São José, the annual incidence rate ratio of the post-centralization period (2012–2016) to the pre-centralization period (2006–2011) was 1.009 for both all TB (*p* = 0.158) and pulmonary TB (*p* = 0.247), whereas corresponding values in Florianópolis were 1.014 (*p* = 0.090) and 1.028 (*p* = 0.003) for all and pulmonary TB, respectively. The latter had a statistically significant increase of 2.8% over the 2012–2016 period.

In 2014 and 2015, 207 patients with a culture-confirmed diagnosis of pulmonary tuberculosis were interviewed, 124 patients from Florianópolis and 83 from São José. The sociodemographic indicators and disease outcome information compiled from the interviews are described in Table 2. In relation to the sociodemographic indicators, the only significant differences observed between the two populations were the proportion of patients who use illicit drugs (*p* = 0.02) and the proportion of patients experiencing homelessness (*p* = 0.03), indicators that were predominant in Florianópolis. Regarding the disease outcome, significant differences in successful treatment and treatment default rates were observed between the two municipalities. For patients who started TB treatment in Florianópolis where care was provided by the BHUs, the treatment success rate was 22% lower (95% confidence interval = 9–33%) and the probability of treatment abandonment was 2.41 times higher (95% confidence interval = 1.21–4.78) when compared to patients who started treatment in São José, where care was provided by the outpatient clinic. Conversely, in Florianópolis, a significantly higher proportion of patients started treatment in the early stages of the disease.

**Table 2:** Sociodemographic indicators and primary and secondary outcome of patients with culture-confirmed pulmonary tuberculosis treated in the municipalities of Florianópolis and São José, Southern Brazil, 2014 and 2015

	Variable	Florianópolis N (%)	São José N (%)	*p* ^a^
Sociodemographic indicators	Gender			
	Female	41 (33.1)	30 (36.2)	0.76
	Male	83 (66.9)	53 (63.8)	
	Age group			
	15–24	28 (22.6)	11 (13.3)	0.09
	25–34	40 (32.3)	23 (27.7)	0.49
	35–44	21 (16.9)	20 (24.1)	0.20
	> 45	35 (28.2)	29 (34.9)	0.31
	Level of schooling			
	Primary education	89 (71.8)	50 (60.2)	
	≥ Secondary education	35 (28.2)	33 (39.8)	0.11
	Cigarette smoking			
	Yes	73 (70.9)	53 (63.8)	
	No	30 (29.1)	30 (36.1)	0.39
	Alcohol abuse			
	Yes	34 (34.7)	34 (41.5)	
	No	64 (65.3)	48 (58.5)	0.44
	Use of illicit drugs			
	Yes	45 (45.9)	23 (27.7)	
	No	53 (54.1)	59 (72.3)	0.02
	Experiencing homelessness			
	Yes	16 (12.9)	3 (3.6)	
	No	108 (87.1)	80 (96.4)	0.03
	TB/HIV co-infection	20 (19.8)	16(19.3)	1.00
Primary outcome	Treatment success	82 (66.1)	71 (84.5)	< 0.01
	Default	32 (25.8)	9 (10.7)	< 0.01
	Death	9(7.2)	3 (3.6)	0.37
Secondary outcome	Initiation of treatment			
	≤ 8 weeks	80 (64.5)	46(55.1)	0.01
	9–16 weeks	35 (28.2)	24(29)	0.33
	> 16 weeks	9(7.3)	13 (15.9)	0.38
	Place of diagnosis			
	BHU	67 (54)	30 (36.1)	0.19
	Hospital	52 (41.9)	43 (51.8)	0.83
	Private clinic	5 (4.1)	10 (12.3)	0.02

^a^To test the hypothesis of equal proportions in Florianópolis and São José

## Discussion

Tuberculosis incidence in Brazil decreased from 37.9% in 2006 to 32.4% in 2016 [9]. In the Southern region of the country, where the studied municipalities are located, TB incidence was estimated at 27.4/100,000 population [9]. Florianópolis and São José are neighboring municipalities, have similar municipal Human Development Indices (MHDI) and per capita income, and are among the municipalities with the highest incidence rates of HIV/AIDS in Brazil [10]. In Florianópolis, the mean TB incidence over the 10-year study period was 38% higher (95% confidence interval 27–49%) than in São José. With the exception of 2010, incidence rates have increased over the last 9 years in Florianópolis. Although both municipalities showed increasing pulmonary TB incidence rates in the 2012–2015 period in comparison to the 2006–2011 period, the increase was not statistically significant in São José (*p* = 0.247), as opposed to Florianópolis (*p* = 0.003); increase in pulmonary TB incidence was about threefold higher in Florianópolis (2.8%) than in São José (0.9%). Taken together, these results indicate a putative beneficial effect of centralized versus decentralized TB care in reducing the number of new TB infections.

During the period of decentralized care, São José had annual oscillations in TB and PTB incidence rates, reflecting the lack of standardization in TB care procedures and the high staff turnover in BHUs. Since 2012, TB care has been centralized and is now carried out by a dedicated team led by a pulmonologist. Care is provided in the same facility of the STD/AIDS Counseling and Testing Center (CTC), contributing to the better access of vulnerable populations to TB diagnosis in the municipality. In addition, in 2013, information on TB was broadcast by Brazilian media due to the case of a popular singer who was diagnosed with pleural TB. In 2014, the Ministry of Health launched a campaign to combat TB using the singer’s image, alerting the population about the symptoms of the disease and the importance of attending BHUs for an early diagnosis. The information campaign, although transitory, seems to have sensitized the population about the disease, stimulating the search for diagnosis, and may be associated with the increased incidence observed in 2013–2014.

The expected improvement in treatment success rates did not occur with the adoption of decentralized care; the positive effect expected from having care closer to the place of residence was not confirmed. The best treatment success rates achieved were 74% in Florianópolis and 78% in São José, which are unsatisfactory results even for the previous goals of WHO (85%) [2]. The treatment success rate of new cases in Brazil remained stable at approximately 70% for more than a decade [1]. Among the countries defined as the top 20 in terms of absolute numbers of estimated incident TB cases, Brazil shared with Russia the position of worst treatment success rate in 2015, 71% [1].

Florianópolis maintained significantly higher default rates than São José, also higher than the 11.0% default rate registered in Brazil among new cases in 2015 [9]. Despite the longer distances patients had to travel to reach the care facility, treatment default in São José remained below the national average in 2015. Poor treatment adherence and premature interruption of treatment contribute to a prolonged infectivity and increase the number of people exposed to *M. tuberculosis* [11]. Treatment default has been associated with individual factors, such as low schooling, TB/HIV co-infection, alcohol abuse, illicit drug use, and homelessness [12, 13]. There were no significant differences between the two municipalities in regard to the level of schooling, use of alcohol, and TB/HIV co-infection. However, the proportion of people experiencing homelessness and reporting the use of illicit drugs was significantly higher in Florianópolis. Of note, drug abuse has been strongly associated with treatment failure in similar studies [12, 14]. Therefore, the larger proportion of drug users among TB patients in Florianópolis may have contributed to the high default rates observed in this study.

Regarding health services, the lack of engagement and commitment to patient integration and education in order to promote the cure of the disease has been associated with an early default in TB treatment [15–18]. In the studied scenario, decentralization assigned extra functions to BHU personnel related to the activities of tuberculosis control. In this context, a uniformity of action was not observed among the 70 units. The lack of a scheduled time for medication withdrawal made it impossible to notice patient non-attendance, allowing for days or weeks without treatment. On the other hand, in the centralized care, all patients had a monthly medical appointment scheduled and reported on the tuberculosis record, enabling a more effective follow-up. In addition, during the appointments accompanied by the study, the patients were constantly briefed on the importance of the proper use of medication, giving emphasis on the consequences of treatment non-adherence (therapeutic failure, the emergence of antimicrobial resistance, and death). These factors may have contributed to the lower default rates observed in São José. The impact of an intensive education strategy on treatment compliance has been demonstrated in Bangladesh and Ethiopia [19, 20].

To increase TB treatment success rates, one of the DOTS recommendations is that the patient should be observed by a trained professional during the intake of medication, thus enhancing the bond between patient and health care provider [7]. The increase in adherence to the directly observed treatment (DOT) method is indeed accompanied by higher treatment success rates and lower default rates [21–24]. During the follow-up period of patients in both municipalities, direct observation of medication intake occurred and was recorded only for patients under treatment for multidrug-resistant tuberculosis, and in some cases of therapeutic failure. In general, the patient sought the health unit weekly, biweekly or monthly, and, in some cases, periodic visits by community agents were registered, characterizing a low adherence to the DOT, regardless of the model of care adopted by the municipality. The acceptance and sustainability of the DOT by health care personnel in the daily routine of services requires patient encouragement and sufficient human resources [21, 22]. The adherence to the DOT in both municipalities is necessary to improve the rates of successful treatment.

Conversely, despite the low adherence to DOT in both municipalities, patients from the prospective cohort study (2014–2015) attended in São José, who were followed throughout the period by the same specialized and dedicated team, had an 84.5% treatment success rate. Some authors have shown that good results in treatment success rates can be obtained with a self-administered treatment, reinforcing the idea that a supporting relationship between patient and health care professional can improve treatment results [25, 26]. For this, a trained and committed team is essential. In the decentralized municipality, in which a frequent turnover of professionals in the BHU TB programs was observed, a low rate of treatment success (66.1%) was reported. Other studies have shown that decentralized TB care is generally characterized by work overload for health care professionals in a scenario of dispersed actions and high staff turnover [21, 27].

The distance from the patients’ place of residence to the place of health care and medication dispensation acts as a barrier to treatment adherence, [15–17, 28] and, therefore, decentralization may be a solution to this problem. The results observed in the present study, however, indicated that, in relation to treatment adherence, the maintenance of a trained and dedicated staff and the education of TB patients were more relevant than the distance to health care services. Characteristics of the studied municipalities, such as their territorial extension and predominance of urban areas may have contributed to the observed results. For rural areas and isolated/hard-to-reach communities, a “virtual” model of care has shown good results, in which the local health professional is oriented and supervised by a specialized group (public health nurses, infectologists, and pulmonologists) [29].

The operational indicators (proportion of smear testing in the second month of therapy, HIV testing, and imaging examinations performed for diagnosis and lesion progression follow-up) were significantly lower in Florianópolis, showing less effectiveness when compared to the centralized care and corroborating the low rates of treatment success and increased TB incidence observed in Florianópolis.

Over the 10-year study period, 71.0% of TB patients were tested for HIV in Florianópolis and 95.7% in São José, with co-infection rates of 21.6% and 22.3%, respectively; co-infection rates of these municipalities were higher than the national average rate (13%) [1]. As of the centralization of TB care in 2012, the municipality of São José integrated tuberculosis control and HIV monitoring services, which started to operate in the same facility, achieving a 68.7% success rate in TB treatment among co-infected patients, in comparison to the 39.1% success rate observed in Florianopolis for the same patient group in the same period. Other studies have shown that this integration results in an increase in treatment efficacy for co-infected individuals, prolonging their survival and maximizing resources [30, 31], and this strategy should be prioritized in low-income areas and/or areas with a high incidence of HIV infection.

Among the positive aspects associated with decentralization, the present study showed a significantly higher proportion of patients initiating treatment within 8 weeks after the onset of symptoms in Florianópolis, where diagnosis took place mostly in the BHUs (54%). Likewise, the proportion of culture-confirmed pulmonary TB cases was 40% higher in Florianópolis than in São José, an indicator that began to improve in 2010 when the Municipal Laboratory started to perform culture tests for all samples. Prior to the adoption/implementation of the rapid molecular detection, the Brazilian Ministry of Health recommended smear microscopy for the diagnosis of TB in patients with respiratory symptoms, indicating culture tests for specific cases only [7]. The São José Municipal Laboratory started to perform culture tests for all samples, as of 2012. However, the BHUs that use the São José Municipal Laboratory service were responsible for only 36.1% of the diagnoses performed in the municipality. This explains why the proportion of new PTB cases confirmed by culture remains very low. This study evidenced that the involvement of the BHUs with the tuberculosis program accelerated and increased TB detection. However, a large proportion of diagnoses still occurred in hospitals, 41.9% in Florianópolis and 51.8% in São José, characterizing the occurrence of late diagnoses and more severe cases of the disease. It is, therefore, necessary to intensify the active search for individuals with symptomatic respiratory diseases, screening people who have contact with TB patients and other vulnerable groups, as well as by promoting joint actions to increase the detection of community cases in the two municipalities.

The variation of the epidemiological indicators of the tuberculosis control programs observed in the 10-year period shows a lack of systematization and monitoring of the control strategies adopted by the municipalities. The observed discrepancy between treatment success rates reported on the Information System (TABNET/DIVE) and those reported in the patients’ records suggests a negligence in feeding data to the monitoring system. Successful campaigns for disease elimination are characterized by locally adapted responses evaluated through consistent local data [32]. Recording and evaluating the outcome of every patient is an integral part of the control program and an excellent tool to evaluate the results of interventions [33]. By knowing the key points, the municipality can focus on specific targets to stop transmission, such as prioritizing high-risk populations [34].

Recently, the Brazilian government has made significant investments to improve the diagnosis of new TB cases, with the implementation of molecular methodologies in the public network, a faster and more sensitive diagnosis. However, detecting, treating, curing, and increasing treatment adherence continue to be great challenges for Brazilian municipalities, requiring investments in personnel and management, strategic planning, and supervision to improve local public policies. Strategies should be thought out, planned, and monitored locally, tailoring the models to the individualized realities. In this context, the present study concludes that the process of treatment decentralization to the BHUs, alone, did not positively influence the main epidemiological indicators, related to the control of tuberculosis, after 10 years of its implementation. In both studied municipalities, strategies should be adopted to address the issue of treatment adherence.

## Conclusion

Decentralization of TB care did not improve the main epidemiological indicators related to TB control. The analysis of TB health service indicators also showed that centralized care had a better performance than decentralized care. Conversely, decentralization improved early diagnosis indicators. Supervision of TB care by a specialized dedicated team ensures that experienced personnel deliver quality service to patients, increasing adherence to control strategies and equity of care. However, the commitment of all levels of health care and the involvement of the community are essential for the effective control of TB.

## Abbreviations

BHU: Basic Health Units
DIVE/SC: Santa Catarina Epidemiological Surveillance Department
DOT: Directly Observed Treatment
DOTS: Directly Observed Treatment Short-Course
GDP: Gross Domestic Product
MHDI: Municipal Human Development Index
PNCT: National Tuberculosis Control Programme
SINAN: National Information System for Notifiable Diseases
SPSS: Statistical Package for Social Sciences
TB: Tuberculosis
WHO: World Health Organization
